# The practice of Burch Colposuspension versus Mid Urethral Slings for the treatment of Stress Urinary Incontinence in developing country

**DOI:** 10.12669/pjms.37.5.4017

**Published:** 2021

**Authors:** Saida Abrar, Lubna Razzak, Raheela Mohsin

**Affiliations:** 1 Dr. Saida Abrar Clinical Fellow Urogynecology and Pelvic Reconstructive Surgery, Department of OBGYN, Aga Khan University Hospital Karachi, Karachi, Pakistan; 2 Dr. Lubna Razzak Clinical Fellow Urogynecology and Pelvic Reconstructive Surgery, Department of OBGYN, Aga Khan University Hospital Karachi, Karachi, Pakistan; 3 Dr. Raheela Mohsin Rizvi Associate Professor Urogynecology, Department of OBGYN, Aga Khan University Hospital Karachi, Karachi, Pakistan

**Keywords:** Burch colposuspension, Tension free vaginal tape, Transobturator tape, Long-term results, Stress urinary incontinence

## Abstract

**Objectives:**

To compare the effectiveness and complications of Burch colposuspension and Mid Urethral Slings (MUS) for the treatment of Stress Urinary Incontinence (SUI).

**Methods:**

We conducted a cross-sectional study of 162 patients who underwent surgery for SUI with Burch colposuspension (n=40), tension free vaginal tape (TVT) (n= 59) or transobturator tape (TOT) (n=63), from 2006 to 2014 at the Aga Khan University Hospital- Karachi. All three groups were assessed in terms of demographics, cure rates, intraoperative and postoperative complications at one and five years using incontinence impact questionnaire-short form-7 (IIQ-7) and urogenital distress inventory -short form-6 (UDI-6).

**Results:**

Mean age of the participants in Burch, TVT and TOT group was 44.1 ± 7.4, 48.3 ± 8.9, 53.0 ± 9.4 respectively. Majority of patients in TVT group were premenopausal (59.3%) and postmenopausal in TOT group (53.9%). Most abdominal hysterectomies were done in Burch group (40) while vaginal hysterectomies and anterior and posterior colporrhaphy in TOT group (55). All the procedures had both subjective and objective cure rate of more than 82% at one year, with TVT having the highest success rate of 96.61%. The objective cure rate in Burch, TVT and TOT group at five years was 74.19%, 90.30% and 81.25% respectively. Intraoperative complications included hemorrhage in one patient during Burch procedure and bladder perforation in two cases of TVT, with no significant difference in short or long-term complications with either procedure.

**Conclusions:**

All the three procedures have equal efficacy and complication rates. Even though TVT is the new gold standard but in view of current debate regarding mesh related complications, there is a need to readdress Burch colposuspension for treatment of SUI.

## INTRODUCTION

International Urogynecological Association (IUGA)^1^ defines SUI as involuntary loss of urine on effort causing a rise in abdominal pressure. It has a severe impact on the quality of life (QOL) of a significant number of women.^2^ Among adult women, its prevalence varies from 12.8% to 46.0%.^3^

After failed conservative management, surgery is considered a standard treatment 4 with the aim to stabilize urethrovesical junction. Various surgical methods are performed but the ideal procedure has still not been defined. Burch colposuspension described in 1961, as a gold standard surgical treatment of SUI in terms of efficacy and safety.^5^ Later TVT became the new gold standard, because of being minimally invasive but equally good success rates.^6^ In 2001, Delorme introduced the TOT procedure with the aim to avoid bladder, bowel, and major vascular injuries, reported with other retro-pubic sling techniques.^7^

Numerous studies have compared the efficacy of Burch colposuspension and MUS. A meta-analysis of colposuspension and MUS demonstrated significantly higher overall and objective cure rates with MUS but a higher risk of bladder perforation in retropubic slings as compared to TOT.^8^ A regional study from Turkey showed that TOT is associated with improved objective cure rates of SUI and QOL.^9^ Similarly a local study from Pakistan revealed that TVT is an effective and safe procedure for the surgical treatment of SUI.^10^

The use of MUS in Asia is less than Western countries. In India MUS is used by about 60% surgeons as primary surgery for SUI while Burch colposuspension is the second choice among the other Asian countries^11^, however there is no study done in Pakistan, which has compared all the three procedures.

Since the Food and Drug Administration Public Health issued a Notification regarding safety of transvaginal mesh in 2011^12^, there has been debate regarding safety of MUS procedures. There is a dire need to readdress the use of Burch colposuspension, which has now become a forgotten skill for surgical treatment of SUI. This study was aimed to compare Burch colposuspension with MUS in terms of efficacy and complication rates.

## METHODS

We conducted a cross-sectional study of 162 women, aged 30 to 70 years who underwent either Burch colposuspension, TOT or TVT for diagnosed SUI at the Aga Khan University Hospital. ICD-9-CM procedure codes were used to determine these procedures, from January 2006 till December 2014. At our hospital, standard definition and classification for the urodynamic studies (UDS) and SUI grades are used in compliance with ICS/IUGA terminology.

All three groups were compared in terms of demographic and clinical characteristics (age, body mass index (BMI) and parity), concomitant surgical procedure, type of surgical procedure, short and long term complications. All patients were evaluated by history, Urogynecological clinical examination, urinalysis and urine culture. The type of SUI was established on basis of clinical symptoms, objective stress test (the test was considered positive if patient leaked urine in supine position on coughing after their bladder were filled with 300 ml of saline) and UDS. UDS was performed by urodynmicist nurse, before surgery in all cases of Burch colposuspension to rule out intrinsic sphincter deficiency (ISD) while in a few cases of mixed urinary incontinence to exclude neurogenic bladder preoperatively. All women with neurogenic bladder, history of anti-incontinence surgery, pregnancy, patients on antipsychotic medications and those who were lost to follow-up were excluded from the study.

The same surgeon performed all the procedures. Foley catheter was retained for 24 hours after MUS group and 36–48 hours postoperatively in the Burch group.

All patients were followed-up at 2 and 6 weeks, 6 months, 1 year, 5 years and thereafter data was collected by telephonic conversation. Post operatively all patients were re-evaluated using incontinence impact questionnaire-short form-7 (IIQ-7) and urogenital distress inventory -short form-6 (UDI-6) for improvement in health-related QOL. Response to each item was originally rated between 0 (not affected at all) and 3 (greatly affected). Postoperatively the patients were regarded as cured if they had negative stress test results and if QOL assessed using IIQ-SF 7 scores showed >90% improvement and improved if the patient was having > 75% improvement in symptoms.^13^

The intraoperative and postoperative complications like bowel and bladder injury, hemorrhage, urinary retention, hematoma, prolonged hospital stay, voiding dysfunction and mesh related complications like exposure, erosion, infection and rejection were recorded for all patients.

Entry of data and analysis were done using SPSS version 21. The data from the various groups (Burch colposuspension, TVT, TOT) were compared using the chi-squared test for categorical variables, and ANOVA for parametric quantitative variables. P-value < 0.05 were considered statistically significant.

### Ethical approval

Ethical approval for this study was taken from institutional Ethical Review Committee (5017-Obs-ERC -17).

## RESULTS

Our study evaluated 63 patients in TOT, 59 patients in TVT and 40 patients in the Burch colposuspension group. There was no statistically significant difference in baseline demographics and clinical characteristics among the three groups other than age and menopausal status (Table-I). The only confounding variables were total abdominal hysterectomy and bilateral salpingectomy performed in all cases of Burch colposuspension, concomitant anterior and posterior colporraphy performed with TVT, TOT and few cases of Burch colposuspension and vaginal hysterectomy in certain cases of TOT and TVT (Table-II). It is a routine practice in our unit to perform Burch colposuspension for SUI, in whom abdominal hysterectomy is planned for gynecological conditions.

**Table-1:** Demographic and Clinical Characteristics of Study Population=n=162.

Parameter	Burch (n=40)	TVT (n=59)	TOT (n=63)	P value
Age, years	44.1 ± 7.4	48.3 ± 8.9	53.0 ± 9.4	0.001
Parity	4.4 ± 2.1	4.3 ± 2.4	4.3 ± 2.2	0.991
Body mass index (kg/m2)	31.1 ± 8.0	28.7 ± 4.7	30.3 ± 5.5	0.116
Mean follow-up (months)	38 (30-60)	36.5 (28-60)	36.7 (28-60)	0.11
***Comorbidity***				
Hypertension	12 (30.0%)	18 (30.0%)	24 (38.0%)	0.590
Diabetes mellitus	8 (20.8%)	17 (28.8%)	22 (34.9%)	0.305
Pulmonary disease	10 (25.0%)	20 (33.8%)	16 (25.0%)	0.400
Constipation	5 (12.5%)	7 (11.8%)	16 (25.3%)	0.092
***Menopausal status***				
Premenopausal	15 (37.5%)	35 (59.3%)	13 (20.6%)	0.000
Postmenopausal	4 (10.0%)	21 (35.5%)	34 (53.9%)	

***Abbreviations:*** TOT, transobturator tape; TVT, tension free vaginal tape, Data are mean ± SD or n= (%).

**Table-II:** Details of Procedure and Type of Incontinence.

Procedures	Burch (n=40)	TVT (n=59)	TOT (n=63)
Anti-incontinence surgery alone	0 (0)	11 (18.6%)	2 (3.1%)
Vaginal Hysterectomy	0 (0)	26 (44.0%)	55 (87.3%)
Abdominal Hysterectomy	40 (100%)	21 (35.6%)	5 (7.9%)
AP repair	3 (7.5%)	33 (55.9%)	44 (69.8%)
Mean duration of catheterization	3.9 ± 2.2	2.2 ± 0.7	1.8 ± 1.3
Urodynamic studies	40 (100%)	50 (84.7%)	29 (46.0%)
ISD	0 (0)	12 (20.3%)	12 (19.0%)
MUI	4 (10.0%)	7 (11.8%)	26 (41.2%)

***Abbreviations:*** TOT, transobturator tape; TVT, tension free vaginal tape; ISD, Intrinsic sphincter deficiency; MUI, Mixed urinary incontinence; AP repair, Anterior and Posterior repair.

The incidence of perioperative complications are sown in Table-III. Only one patient had intraoperative hemorrhage during Burch procedure and 2 patients had intraoperative bladder perforation during TVT (2.5%). There were no significant short or long-term complications with either procedure.

**Table-III:** Surgical and Functional outcomes in the Study Patients

Outcome	Burch (n =40)	TVT (n =59)	TOT (n =63)	P value
***Intra-Operative complications***				
Hemorrhage	1 (2.5%)	0 (0)	2 (3.1%)	0.364
Bladder injury	0 (0)	2 (3.3%)	0 (0)	0.192
Bowel injury	0 (0)	0 (0)	0 (0)	0
Neurovascular injury	0 (0)	0 (0)	0 (0)	0
***Postoperative early complications (within 7 days of surgery)***				
Prolong length of hospital stay	2 (5%)	1 (1.69)	0 (0)	0.212
Perineal hematoma	0 (0)	0 (0)	1 (1.58)	0.441
Extended antibiotics	5 (12.5%)	2 (3.3%)	5 (7.9%)	0.056
Shift to special care	1 (2.5%)	0 (0)	0 (0)	0.025
Urinary retention	5 (12.5%)	2 (3.3%)	0 (0)	0.152
***Postoperative late complications (>2 weeks of surgery)***				
Wound infection	3 (7.5%)	0 (0.0)	0 (0)	0.063
Mesh Exposure	0 (0)	2 (3.4)	4 (6.3%)	0.196
De novo Urgency	1 (2.5%)	1 (1.7%)	0 (0)	0.531
Short-term Voiding Dysfunction	2 (5%)	1 (1.7%)	0 (0)	0.212
***Compartment defects > one year***				
Enterocele	1 (2.5%)	0 (0)	0 (0)	0.235
Cystocele	2 (5%)	2 (3.4%)	3 (4.7%)	0.866

***Abbreviations:*** TOT, transobturator tape; TVT, tension free vaginal tape.

Statistical tests: chi-squared and ANOVA. Data are n/N (%) unless otherwise specified.

The subjective and objective cure rates of SUI after one and five years are shown in Table-IV. There was no significant difference in the subjective and objective cure rates of SUI for all these procedures both at one and five years follow-up. For analysis of cure rates at the end of five years, 32 patients were available in the TOT group, 42 in TVT group and 31 in the Burch group. Seven patients in the TOT group were incontinent by the end of 1 year: medical therapy was commenced in four patients while three patients were re-operated on and TVT procedure was performed. The TVT slings were placed without removing TOT sling. De novo urge incontinence developed in one patient in the TOT group. Ten patients were incontinent in the Burch group: six patients received medical treatment while four patients were re-operated on with the TVT procedure. Urinary retention was observed in one patient in the TOT group.

**Table-IV:** Cure rates on Stress Urinary Incontinence (SUI) at 1 and 5 years.

Variable	Burch (n=40)	TVT (n=59)	TOT (n=63)	p-value
Cure rates at 1 year				
Subjective	34/40 (85.0%)	57/59 (96.61%)	55/63 (87.30%)	0.1
Objective	33/40 (82.5%)	56/59 (94.91%)	54/63 (85.71%)	0.12
Cure rates at 5 years				
Subjective	24/31(77.41%)	39/42 (92.85%)	27/32 (84.37%)	0.17
Objective	23/31(74.19%)	38/42 (90.30%)	26/32 (81.25%)	0.18

***Abbreviations:*** TOT, transobturator tape; TVT, tension free vaginal tape

Values are given as number (percentage) unless otherwise indicated.

## DISSCUSION

Our study showed almost similar objective and subjective success rates and complications with all the three procedures for treatment of SUI at one and five years follow-up. This is in contrast to a meta-analysis,^14^ which showed that MUS had significantly higher overall and objective cure rates compared with Burch procedures (odds ratios [OR] 0.59 and 0.51, respectively; both p < 0.0003).

A retrospective study of Burch colposuspension (n = 498) vs. TOT (n = 272) showed similar five year cure rate for both groups, however, fewer patients developed De-novo urgency or required self-clean intermittent catheterization (CISC) in TOT group than Burch colposuspension.^15^

The current study showed 3.4% risk of bladder perforation only in TVT group while nine trials comparing these three procedures found bladder perforation of 6% in TVT versus 1 % in open colposuspension (RR 4.24, 95% CI: 1.71--10.52).^16^ The risk factors associated with bladder perforation in our study were same as reported in literature i.e., high BMI and previous pelvic surgery.^17^,^18^

We found 5% Voiding Dysfunction (VD) in Burch and 1.7% in TVT group, similar to results observed in a recent systematic review.^14^ A similar retrospective study found 6.1% short term VD with TVT and 7.9% with Burch^16^ but among the MUS; TVT was associated with long-term-VD.^19^ However we did not observe any long-term VD in MUS requiring tape resection, removal or CISC. In line with the current study, a recent randomized trial showed a small, non-significant difference in VD (21.8% versus 27.6%) in cases of TOT versus TVT respectively.^20^ This fact may be explained by the horizontal position of the sling that minimized the possibilities of urethral compression.

The frequency of de-novo urgency urinary incontinence (UUI) was higher in colposuspension than in MUS which is similar to long-term follow up study reporting de novo UUI ranging from 15-41% after Burch 18, 4-17 after TVT 20-22 and 1.5- 6.7% with TOT.^20^

We did not observe any long-term mesh related complication, however asymptomatic tape exposure was seen with TVT (3.4%) and TOT (7.1%) but there was no statistically significant difference. Studies on MUS have reported mesh related long-term complication including vaginal tape erosion/extrusion rate as 1.5% and 0.4% with TVT and TOT respectively and urethral erosions and sexual dysfunction like dyspareunia or hispareunia after MUS.^22^-^24^ Our study showed 2.5% enterocele and 5% cystocele at five years follow-up with Burch colposuspension while literature shows it in range of 1-34%.^25^

### Strength of the study

The strength of our study includes long term follow up, the exclusion of ISD for Burch colposuspension by prior UDS and one surgical hand.

### Limitations of the study

The limitations of study include the retrospective data of one center with small number of patients in each group. We performed Burch colposusspension only for urethral hypermobility and hence results for ISD cannot be compared.

## CONCLUSION

The surgical outcome of these three procedures is comparable with almost similar complications and cure rates of SUI. In the current era of controversy regarding the use of mesh and its associated complications, Burch colposuspension could be a useful alternate for the surgical treatment of SUI.

### Authors Contribution

**SA:** Conceived, manuscript writing and editing. is responsible for integrity of the study.

**LR:** Data collection and did statistical analysis

**RR:** Did review and final approval of manuscript.
